# In Vivo Antidepressant Effect of *Passiflora edulis* f. *flavicarpa* into Cationic Nanoparticles: Improving Bioactivity and Safety

**DOI:** 10.3390/pharmaceutics12040383

**Published:** 2020-04-21

**Authors:** Jovelina Samara Ferreira Alves, Alaine Maria dos Santos Silva, Rodrigo Moreira da Silva, Pamella Rebeca Fernandes Tiago, Thais Gomes de Carvalho, Raimundo Fernandes de Araújo Júnior, Eduardo Pereira de Azevedo, Norberto Peporine Lopes, Leandro De Santis Ferreira, Elaine Cristina Gavioli, Arnóbio Antônio da Silva-Júnior, Silvana Maria Zucolotto

**Affiliations:** 1 Research Group on Bioactive Natural Products (PNBio), Laboratory of Pharmacognosy, Graduate Program in Health Sciences, Federal University of Rio Grande do Norte (UFRN), Natal 59.012-570, Brazil; jsfa.farma@gmail.com; 2 Laboratory of Pharmaceutical Technology & Biotechnology (TecBioFar), Graduate Program in Pharmaceutical Sciences, Pharmacy Department, Federal University of Rio Grande do Norte (UFRN), Natal 59.012-570, Brazilarnobiosilva@gmail.com (A.A.d.S.-J.); 3 Nucleus Research in Natural and Synthetic Products (NPPNS), Department of Physics and Chemistry, Faculty of Pharmaceutical Sciences of Ribeirão Preto, University of São Paulo, Ribeirão Preto 14.040-903, Brazil; rodeira@gmail.com (R.M.d.S.); npelopes@fcfrp.usp.br (N.P.L.); 4 Laboratory of Behavioral Pharmacology, Department of Biophysics and Pharmacology, Federal University of Rio Grande do Norte (UFRN), Natal 59.078-970, Brazil; f.pamellat@gmail.com (P.R.F.T.); egavioli@hotmail.com (E.C.G.); 5 Graduate Program in Health Sciences, Departament of Morfology, Federal University of Rio Grande do Norte (UFRN), Natal 59.078-970, Brazil; thaisbida2011@hotmail.com (T.G.d.C.); araujojr.morfologia@gmail.com (R.F.d.A.J.); 6 Graduate Program of Biotechnology, Laureate International Universities—Universidade Potiguar (UnP), Natal 59.056-000, Brazil; eduardo.azevedo@unp.br; 7 Laboratory of Quality Control of Medications (LCQMed), Pharmacy Department, Federal University of Rio Grande do Norte (UFRN), Natal 59.012-570, Brazil; lean_sf@yahoo.com.br

**Keywords:** flavonoids, nanotechnology, depression, oral toxicity, PMMA, Eudragit

## Abstract

A variety of neuroactive flavonoids can be found in the species of the *Passiflora* genus; however, their difficulty in crossing the blood–brain barrier limits their in vivo neuropharmacological activity. In this study, cationic nanoparticles were developed as a novel nanocarrier for improving the antidepressant activity of *Passiflora edulis* f. *flavicarpa* leaf extract. Formulations obtained using Eudragit E PO polymethylmethacrylate copolymer, as polymeric matrix had their physicochemical properties investigated. The analytical content of the flavonoids vicenin-2, orientin, isoorientin, vitexin, and isovitexin was determined in the plant extract. Small-sized and spherical nanoparticles loaded with *Passiflora edulis* f. *flavicarpa* were obtained with positive zeta potential and high encapsulation efficiency. In addition, the nanosystems were shown to be stable for at least 6 months. The antidepressant activity of *P. edulis* extract (50 and 100 mg/kg) as well as the extract-loaded nanoparticles (5 mg/kg) were investigated in mice using the forced swimming test, where the latter increased the potency of the former by 10-fold. In addition, histopathological and biochemical analysis confirmed the biocompatibility of the extract-loaded nanoparticles. This study demonstrated that the Eudragit cationic nanoparticles were able to improve the antidepressant activity of *P. edulis* in the central nervous system of mice.

## 1. Introduction

Depressive disorders are the major cause of deaths by suicide around the world. According to the World Health Organization (WHO), depression will be the most common disease in the world by 2030 [1]. The classical drugs used to treat depression cause many side-effects and, therefore, have been associated with poor patient compliance. Thus, new alternatives have constantly been investigated with the purpose of discovering new antidepressants with fewer side effects [2,3,4]. Previous reports have demonstrated the antidepressant potential of extracts of herbal plants, which have been formerly used for other diseases due to their antioxidant properties [5,6,7,8]. Such potential antidepressant activity has been attributed to a protective effect against neuroinflammation and oxidative stress associated with these plants [3,5,9].

*Passiflora* is the largest and most important genus of the Passifloraceae family, comprising over 400 species [10]. These species have both medicinal and agronomic relevance. In traditional medicine, its leaf infusions have been used as tranquilizer/sedative in many countries [2,10,11,12]. *Passiflora edulis* f. *flavicarpa*, popularly known as “yellow passion fruit” is largely cultivated in South America, with Brazil being the top world producer [12,13]. Phytochemical constituents of leaves include flavonoids, saponins, cyanogenic glycosides, and terpenoids [10,14]. Recently, behavioral studies in mice have shown that the leaf extract of *P. edulis* f. *flavicarpa* displayed antidepressant-like activity, suggesting that this effect was correlated with the presence of *C*-glycosyl flavones [3,11,15,16,17].

The therapeutic use of natural compounds requires an efficient extraction process that is able to provide enough phytoactive compounds that are necessary to overcome the biological barriers and induce the desired pharmacological response [18,19]. Nano-delivery systems have shown interesting and promising properties for enhancing the activity of natural compounds [18,19]. These small particles could have the ability to overcome the blood–brain barrier and enhance drug targeting to the central nervous system (CNS) [20,21]. More than 98% of the small drug molecules and almost 100% of macromolecules (>500 Da) are unable to reach therapeutic concentration in the CNS [20]. Among the colloidal nanocarriers potentially able to solve this problem, polymeric nanoparticles stand as an innovative and effective approach to enhance the transport of drugs to the CNS [18,19,20,21,22,23,24].

These nanoparticles should be biocompatible and not induce any toxic or relevant inflammatory response to the human body [24,25]. The Eudragit E PO is a cationic polymethylmethacrylate (PMMA) copolymer accepted by several regulatory agencies around the world for oral and topical administration of drugs. It has shown biocompatibility, bioadhesivity, efficient adsorption, and increased permeability [25,26]. In previous studies, Eudragit nanoparticles have shown to decrease the toxicity and improve the efficacy of flavonoids [24,27]. The swelling and cationic properties of copolymer allows high encapsulation efficiency of natural compounds [18,23,24]. This concept can be applied to other cationic polymers or cationic-functionalized nanoparticles for parenteral administration of drugs and natural products.

Molecules of flavonoid *O*-glycosides need to be hydrolyzed in the stomach’s acidic pH in order to be absorbed in the gastrointestinal tract. The *C*-glycosyl flavonoids, such as the antidepressant bioactive compounds from *P. edulis* [3,15,16,17], are poorly absorbed in the gastrointestinal tract [7,12,13] because they need to be previously metabolized by the intestinal microbiota [7]. This fact, associated with the low aqueous solubility of aglycones, short elimination half-life, and rapid in vivo elimination of flavonoid glycosides, limits their clinical application [7,24,28]. Thus, nano-delivery systems show interesting and specific properties that can be used to improve the efficacy of these neuroactive flavonoids [24,29,30].

To date, the majority of flavonoid glycosides are extracted from plant material, whose low yield requires multiple laborious steps [13,16,31]. In fact, the synthesis of *C*-glycosyl flavonoids is an exhaustive, expensive, and unsustainable process [32]. For these reasons, investigating the therapeutic application of flavonoid-enriched extract seems to be more promising [2,5,6,12,13]. Therefore, this current study aims to prepare and characterize *P. edulis* f. *flavicarpa* extract-loaded Eudragit nanoparticles, whose antidepressant-like effects and biocompatibility were investigated in mice.

## 2. Materials and Methods

### 2.1. Materials

The copolymer of polymethylmethacrylate Eudragit E PO (EUD), was purchased from Pharma Polymers (Röhm GmbH & Co. KG, Darmstadt, Germany). Polyvinyl alcohol (PVA) with a viscosimetric molecular mass of 4.7 × 104 g/mol was purchased from Vetec (São Paulo, Brazil). Ethanol (EtOH) was purchased from Labsynth (São Paulo, Brazil). Purified water (1.3 μS) was obtained using a reverse osmosis equipment OS50 LX (Gehaka, SP, Brazil). HPLC-grade acetonitrile and methanol were purchased from J. T. Backer (Mexico City, Mexico). Formic acid 98%–100% used for preparing the mobile phase was of analytical grade (Proquimius, Rio de Janeiro, Brazil). The ultrapurified water used for HPLC was obtained using a Milli-Q system (Millipore, Bedford, MA, USA). The mobile phases were filtered through polyvinylidene fluoride (PVDF) solvent filters (0.45 μm) (Merck; Milan, Italy). Vials and PVDF syringes were acquired from Analitica (São Paulo, Brazil). The reference standards vitexin (≥96%), luteolin (≥98%), and isovitexin (≥98%) were purchased from Sigma-Aldrich (St. Louis, MO, USA). Orientin and isoorientin were isolated from *P. edulis* f. *flavicarpa* leaves collected in Antônio Carlos, Santa Catarina, Brazil and a voucher specimen was deposited in the Herbarium at the Federal University of Santa Catarina university (FLOR 33886). The flavonoids was identified by LCQ/TOF mass spectrometry (Agilent Technologies, Santa Clara, CA), whose purity was confirmed by Shimadzu UFLC-XR™ system (Shimadzu, Kyoto, Japan), in São Paulo University, Ribeirão Preto-Brazil. Vicenin-2 isolated from *P. edulis* f. *flavicarpa* leaves was provided by Prof. Dr. Josean Fechine Tavares, Federal Universisty of Paraíba, João Pessoa-Brazil.

### 2.2. Preparation of the Leaf Extract

The leaves of *Passiflora edulis* f. *flavicarpa* Degener (Passifloraceae) were collected at Gurjaú plantation located in Coronel Ezequiel city, Rio Grande do Norte State, Brazil (6°23′44.2′′ S, 36°75′10′ 23.7′′ W) on 17 May 2017. A specimen voucher was deposited at the Herbarium of the Federal University Rural of Rio Grande do Norte—UFERSA (identification number: 13751.6). This project was previously authorized for collecting (SISBIO 5524549) and accessing biodiversity for research purposes (SISGEN A618873). The leaves were dried in an air circulation oven at 45 °C for 72 h. The extract was obtained by turbo-extraction using 7.5% (*w/v*) drug material content and 60% (*v/v*) hydroethanolic solution for 5 min. The hydroethanolic extract (EP) was concentrated at reduced pressure at 35 °C using a rotary evaporator R3 Buchi (Konstanz, Germany) and lyophilized in a freeze drier L101 Liotop (Liobras; São Carlos, Brazil) for 48 h.

### 2.3. Standardization of Leaf Extract by LC-QqQ-MS/MS

The content of vicenin-2, orientin, isoorientin, vitexin, and isovitexin in the *Passiflora edulis* f. *flavicarpa* leaf extract was determined using a Preminence liquid chromatographic system (Shimadzu, Kyoto, Japan) equipped with an online degasser DGU-20A3R, a solvent pump LC-20AD, an autosampler SIL-20ACHT, a column oven CTO-20AC, and a controller CBM-20A, coupled with a triple quadrupole mass spectrometer (MS) API 3200 (AB Sciex, Concord, Ontario, Canada) (LC-MS/MS). The chromatographic separation was achieved using an Ascentis express C_18_ column 100 × 4.6 mm, 2.7 µm (Supelco, Bellefone, PA, USA) and an equivalent guard column (5 mm; Phenomenex, Torrance, CA, USA) maintained at 40 °C. The mobile phase was formic acid 0.1% (A) and methanol (B) eluted in a gradient mode. Mixed standard stock solution of five reference compounds was prepared in 50% MeOH (*v/v*) and diluted to a final concentration range of 5–500 ng·mL^−1^. The volume of injection was 5 µL and the flow rate was 1.0 mL·min^−1^ with splitter. The MS was operated in the negative ion and multiple reaction monitoring (MRM) mode for the transitions 593 → 353 (vicenin-2), 447 → 327 (orientin and isoorientin), 431 → 311 (vitexin and isovitexin), and 285 → 133. Prior to the analysis, the extract samples (60 ug·mL^−1^) were diluted in the mobile phase and filtered through a polytetrafluoroethylene membrane with a pore size of 0.22 µm.

The calibration curves used for quantifying the compounds was linear over the concentration range of 5.0–500 ng·mL^−1^ (Figures S1–S5). Lower limits of detection and quantification were determined. Precision and accuracy, which were assessed within and between runs, were achieved with relative standard deviations and relative errors below 5% for all concentrations evaluated. Carryover from subsequent injections was not observed. Validation of the analytical procedures was performed according to the ICH guidelines for validation of analytical methods, whose analyses were performed in triplicate for each concentration, and the data were expressed as mean ± standard deviation (SD) [33,34].

### 2.4. Preparation of Extract-Loaded Nanoparticles

Extract-loaded nanoparticles (NPEP) were prepared by the nanoprecipitation method [25,26]. The organic phase (OP) (6 mL) containing 45 mg of Eudragit E PO and leaf extract (EP) in 1:10, 1:5, and 2:5 EP/Eudragit E PO ratios (4.5, 9.0, and 13.5 mg, respectively), were injected into the aqueous phase (AP) (14 mL) containing 35 mg of PVA as the surfactant agent under magnetic stirring (720 rpm) at 25 °C [25,26]. For preparation of blank nanoparticles (NPB), 0.75% *w/v* Eudragit E PO solution in 10:90 *v/v* (H_2_O/EtOH) was injected into an aqueous solution of 0.25% *w/v* PVA using a burette at 1.0 mL·min^−1^ under magnetic stirring at 720 rpm. The solvent evaporation step was performed at 30 °C using a lyophilizer L101 Liotop (Liobras; São Carlos, Brazil). For preparing the extract-loaded nanoparticles (NPEP), different extract/copolymer weight ratios (1:10, 1:5, and 2:5) were used. The samples were stored in hermetically sealed glass flasks at 25 °C until analysis. All experiments were performed in triplicate and the data were expressed as mean ± SD.

### 2.5. Characterization of Extract-Loaded Nanoparticles

#### 2.5.1. Particle Size and Zeta Potential Measurements

The mean particle size and polydispersity index (PdI) of NPB and of NPEP were measured by photon correlation spectroscopy at 659 nm, with a detection angle of 90° (Nano ZS Zetasizer, Malvern Instruments Corp., Malvern, UK) at 25 °C using polystyrene cuvettes with a path length of 10 mm. The zeta potential was determined by laser Doppler anemometry using the Nano ZS Zetasizer. At least 10 determinations were carried out for each sample diluted to 0.022 wt % with purified water. Data were expressed as mean ± SD.

#### 2.5.2. Extract-Loading Efficiency

The analyses of extract-loading efficiency were carried out on a Shimadzu UFLC-XR system (Shimadzu, Kyoto, Japan) equipped with two LC 20-ADXR solvent delivery units, autosampler (SIL-20ACXR), degassing unit (DGU-20A3), photodiode-array detection (SPD-M20A), and column oven (CTO-20 AC) with a column Shim-pack XR-ODS (particle size 75 × 4.6 mm, 2.2 µm pore size; Shimadzu).

The chromatographic conditions used for the analysis of free extract and encapsulated isoorientin followed the method validated by ultrahigh performance liquid chromatography for quantification of isoorientin in leaf extract of *Passiflora edulis* f. *flavicarpa*. The analyses were carried out in a UHPLC-UV-DAD system for linearity, accuracy, and precision of the method. These data are presented in the Supplementary Materials Table S1 and Figure S6 [35].

The encapsulation efficiency (EE) was calculated by subtracting the free isoorientin in the nanoparticle suspension (Wfree) from the total amount of isoorientin (Wtotal) in the extract using UHPLC-UV-DAD. A total of 20 mL of nanoparticle suspension was subjected to ultracentrifugation at 10.867× *g* for 5 min in a microcentrifuge (NT805, Nova Técnica, Piracicaba, Brazil) [36]. The supernatant was then collected and reduced to 0.9 mL on a rotary evaporator, followed by dilution with 0.9 mL of methanol, and then filtered on 0.22 um PVDF filter. The free extract sample (10 mg/mL) was dissolved in MeOH/H_2_O (1:1) and analyzed by UHPLC-UV-DAD for determination of the isoorientin content. The encapsulation efficiency (*%EE*) was calculated by using Equation (1) below. The analyses were performed in duplicate and the results are presented in the Supplementary Materials Figures S7 and S8.

(1) $$\%EE=\frac{Wtotal-Wfree}{Wtotal}\times 100$$

#### 2.5.3. Atomic Force Microscopy (AFM)

The shape and surface of NPB and NPEP were assessed through AFM images. The dispersions were diluted with purified water at the ratio 1:25 (*v/v*) and dropped over a cover slip, dried under desiccator for 24 h, and then analyzed in an atomic force microscope model SPM-9700 (Shimadzu, Tokyo, Japan) at room temperature with a cantilever non-contact and 1 Hz scanning.

#### 2.5.4. Attenuated Total Reflectance Fourier Transform Infrared (ATR-FTIR)

The interaction between the chemical constituents of the extract and Eudragit nanoparticles was investigated using a ATR-FTIR spectrophotometer SHIMADZU IR Prestige 21 (Shimadzu, Tokyo Japan) [37]. Prior to the analysis, the colloidal dispersion of nanoparticles was concentrated using a vacuum concentrator (Labconco Centrivap, Kansas City, MO, USA) for 7 h. The spectra were obtained at 20 scans with a resolution of 4 cm^−1^ (4000 and 500 cm^−1^) for each individual component (EP, Eudragit PMMA and PVA) as well as the nanoparticles (NPB and NPEP).

#### 2.5.5. Physicochemical Stability

NPEP samples were stored in hermetically sealed flasks at 8 °C for 6 months. At intervals of 7 and 15 days, the particle size as well as zeta potential were determined. The measurements were performed at 25 °C using the parameters previously described in Section 2.5.1. All analyses were performed in triplicate and the data were expressed as mean ± SD.

### 2.6. In Vivo Experimental Procedures

#### 2.6.1. Animals

The biocompatibility analysis (experiment 1) was performed using female Swiss mice (8 weeks of age, 35 ± 5 g), whereas behavioral experiments (experiment 2) were performed using male Swiss mice (12 to 15 weeks of age, 40 ± 10 g) housed at the Federal University of Rio Grande do Norte (Natal, Brazil) following the current guidelines and experimental standardization [38]. The mice were housed in cages (41 × 34 × 16 cm, 10 mice per cage), covered with a sawdust bed under standard conditions (22 ± 2 °C, 12 h light cycle, lights on at 06:00) with water/food ad libitum. A total of 22 animals were used for experiment 1, distributed according to weight, and the experiment was performed in one day. In order to provide more reliability for the obtained results, 91 animals were used for experiment 2 and the tests were carried out on three different days using 3–4 animals per group per day due to the variability of animal behavior. Behavioral procedures were performed between 09:00 and 12:00, and all animals were used only once. The studies were authorized by the Local Ethics Committee for Animal Use of the Federal University of Rio Grande do Norte (license no. 027–2017, 7 November 2017), ARRIVE (Animal Research: Reporting of In Vivo Experiments) guidelines [39], and Brazilian law no. 11.794/2008 for the Care and Use of Experimental Animals.

#### 2.6.2. In Vivo Biocompatibility of EP, NPB, and NPEP

This study was conducted in accordance with the Organization for Economic Cooperation and Development (OECD) Guidelines for Chemical Testing (test number 423) [38]. In the first stage of the experiment, four female rats individually received 5 and 50 mg/kg of free extract (EP), 5 mg/kg of extract-loaded nanoparticles (NPEP), and blank nanoparticles (NPB) by gavage after 8 h of overnight fasting. The animals were observed during 4 h for detection of any of the following signs of toxicity: color change of the eyes and skin; presence of excretion, stool, mucous, and secretions; urination; defecation; autonomic activities; change in posture; gait; convulsions; aggressiveness; and any strange behavior. The animals were monitored for the next 48 h for survival and signs of toxicity. If the animals showed no sign of toxicity and survived for more than 48 h, they received the highest doses of 300 and 2000 mg/kg of EP followed by 48 h of monitoring the signs of toxicity and survival. The second stage was carried out with five females for each group. In the EP group, the highest dose that showed no sign of toxicity was administered. The NPEP and NPB groups remained at the same dose and a saline control group was also included. After gavage, the surviving animals were observed for at least once a day for a total of 14 days. At the end of this period, the animals were anesthetized, 800 µL of blood was collected, and they were euthanized by cervical dislocation. The animals were dissected and organs (brain, kidneys, heart, liver, and spleen) were removed, weighed and fixed in 10% neutral buffered formalin, dehydrated, and dipped in paraffin. Sections of 5 μm thickness were obtained for hematoxylin and eosin (H&E) staining and examination by light microscopy (40×, Nikon E200 LED, Nikon Corporation, Tokyo, Japan). Three sections of each organ (five animals per group) were qualitatively analyzed by two pathologists. Biochemical measurements were performed with approximately 400 µL of serum obtained from blood after centrifugation (1006× *g*; 30 min) using Labmax-240. The biochemical parameters analyzed were albumin (ALB), aminotransferase (ALT), aspartate aminotransferase (AST), urea (UREA), and creatinine (CREA).

#### 2.6.3. Drug Treatment for Behavioral Assays

For the treated groups, EP, NPB, NPEP, and nortriptyline (Novartis Biociências S.A., São Paulo, Brazil) were previously dispersed in saline solution and administered orally (p.o.) at a dose of 10 mL/kg, 90 min before the behavioral experiments. The control group was treated with the same volume of saline solution, as well as the same route of administration as the treated groups.

#### 2.6.4. Forced Swim Test (FST)

This experimental model was performed according to the procedures described by Porsolt et al. [40] in which the mice were forced to swim in a transparent glass cylinder (24 cm in height per 18 cm in diameter) containing 18 cm^3^ of water at 23 °C for 6 min. The immobility time (time spent in water without making any attempt to escape) was recorded by an experienced observer during the last 4 min of a single experimental session. After the behavior assessment, the animals were kept heated using a heating bed until complete drying and then brought back to the cages. All evaluations were recorded using a computerized video camera system.

#### 2.6.5. Open Field Test

The mice’s spontaneous locomotor activity was assessed using an open field apparatus (40 × 40 × 40 cm) with black floor and walls where each animal was placed at the center of the open field whose total traveled distance was recorded (in meters) for 30 min using a video camera interconnected with an automated activity monitoring system (Anymaze, Stoelting Co., Wood Dale, IL, USA). The field was cleaned with 10% ethanol solution after each behavioral evaluation.

#### 2.6.6. Statistical

Experimental values were expressed as mean ± SD. Student’s *t*-test was used for paired comparisons of the analytical data. Univariate analysis of variance (one-way ANOVA) was used for multiple comparisons by Newman Keuls’ test and Student’s *t*-test. Values of *p* < 0.05 were considered to be statistically significant.

## 3. Results and Discussion

### 3.1. Extract Standardization by LC-QqQ-MS/MS

Because the phytochemical composition of a herbal material changes according to the season, the use of a quantified extract of *P. edulis* might ensure the reproducibility and quality of the resulting extract-loaded nanoparticles [10,41]. Therefore, the identification of the site of plant collection and the posterior quantification of the bioactive compounds in the extract are required by most of the pharmaceutical regulatory agencies [41,42].

**Figure 1:**
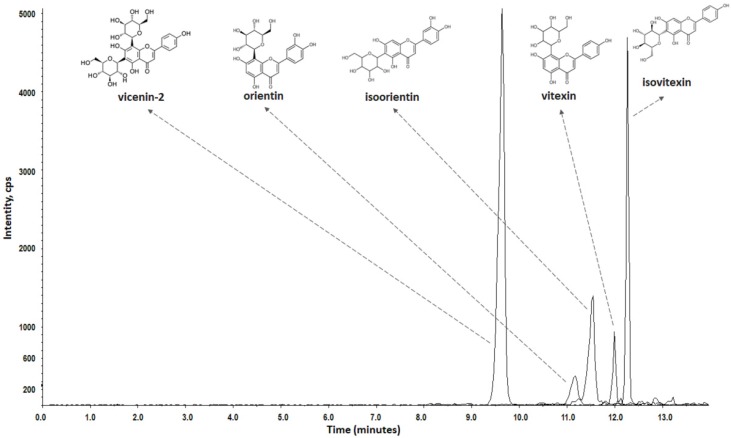
LC-QqQ-MS/MS chromatogram of *Passiflora edulis* f. *flavicarpa* leaf extract. Compounds: vicenin-2 (**1**; *R_t_* 9.8 min); orientin (**2**; *R_t_* 11.3 min); isoorientin (**3**; *R_t_* 11.5 min); vitexin (**1**; *R_t_* 11.9 min), and isovitexin (**1**; *R_t_* 12.3 min); column: Shim-pack XR-ODS (75 × 4.6 mm, particle size of 2.2 µm; Shimadzu); mobile phase: A (0.1% HCOOH) and B (MeCN:MeOH; 6:4; *v/v*), 5% B (0–3 min), 5–15% B (3–8 min), 15–30 B (8–33 min), 30–100% (30–50 min); flow rate: 1 mL·min^−1^.

Previous reports suggest that *C*-glycosyl flavones from *P. edulis* leaves have antidepressant activity [10,13,14]. Thus, standard references for the *C*-glycosyl flavones vicenin-2, orientin, isoorientin, vitexin, and isovitexin were chosen for preliminary screening of *P. edulis* extract. The mass spectra of the extract suggests the presence of these flavonoids [10,14]. However, due to the usual occurrence of isomerism in a variety of flavonoid derivatives from *P. edulis* [14], samples of mixed standards, as well as the extract solution, were individually analyzed by chromatography. Retention times and mass spectra of the extract peaks were compared with those of the reference standards mixture. Therefore, the identification of the five flavonoids in the *P. edulis* extract was confirmed, as depicted in Figure 1.

The chromatographic method allowed the proper separation of the different compounds by the reverse-phase chromatography system. The linearity was adequate for all reference standards (Table 1) and the method was considered accurate and exact [33], as shown in Table 2.

The results show that the chromatographic method was selective for the individual quantification of the compounds present in the extract [33]. The extract quantification revealed the contents of 5.20, 0.59, 2.08, 0.20, and 0.96 mg/g for vicenin-2, orientin, isoorientin, vitexin, and isovitexin, respectively.

### 3.2. Preparation and Characterization of Extract-Loaded Nanoparticles (NPEP)

In this current study, the nanoparticles were prepared by nanoprecipitation method using different extract/Eudragit ratios (Table 3) [43,44]. Among the parameters that are used to characterize nanoparticles, particle size is the most commonly used one, which was the parameter that varied the most according to the different extract/Eudragit ratios [22,26,45,46].

#### 3.2.1. Particle Size, Zeta Potential, and Encapsulation Efficiency

Table 3 shows the influence of the extract/Eudragit ratio on particle size. The 1:10 extract/Eudragit ratio resulted in the smallest particle size (65.6 ± 2.1 nm) and the highest encapsulation efficiency (100%) for isoorientin; therefore, this ratio was selected for further studies. Other extract/Eudragit ratios (1:5 and 2:5) were also investigated, which produced particles smaller than 130 nm; however, these formulations exhibited phase separation after 24 h, impairing further determination of encapsulation efficiency.

The final size of nanoparticles in any biological medium will depend on the formation of protein corona around their surfaces. This fact can be affected by the shape, size, hydrophobicity, charge, and chemical nature of the nanoparticle’s surface [47,48]. In addition, nanoparticle fluid dynamics in blood vessels depend on their size, requiring particles from 10 to 100 nm for intravenous circulation and to cross biological barriers [48,49]. Therefore, because the extract/Eudragit ratio of 1:10 afforded nanoparticles with a mean diameter of 65 nm, they were considered suitable for further studies. The physicochemical properties of the selected extract-loaded nanoparticles were considered to be optimal, as they showed small and uniform particle sizes associated with positive zeta potential, which are critical parameters for improving their ability to overcome the blood–brain barrier and therefore enhancing their pharmacological efficacy in the CNS [21,22,36,45,50]. In addition, cationic nanoparticles might increase gastrointestinal absorption [20,26].

The significant decrease in the size of NPB (120 nm) in comparison with that of NPEP (65 nm) (Table 3) seems to be due to the surfactant property of some of the phytocompounds present in the extract. Previous studies have demonstrated a similar behavior for multidrug-loaded Eudragit nanoparticles containing curcumin-celecoxib [8]. In another study, the encapsulation of the flavonoid dihydromyrictin into Eudragit nanoparticles afforded particles of 146 nm in size [24].

**Figure 2:**
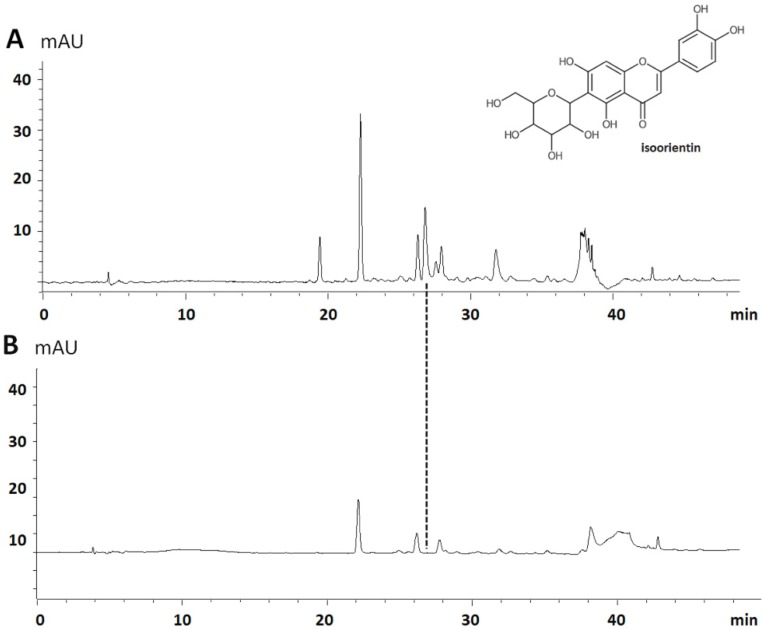
UHPLC-UV-DAD chromatogram of the *P. edulis* f. *flavicarpa* leaf extract, isoorientin compound (**1**; *R_t_* 26.7 min) (**A**), and supernatant of extract-loaded nanoparticles (NPEP) (**B**). Column: Shim-pack XR-ODS (75 × 4.6 mm, 2.2 µm particle size; Shimadzu); mobile phase: A (0.1% HCOOH) and B (MeCN/MeOH; 6:4; *v/v*), 5% B (0–3 min), 5%–15% B (3–8 min), 15–30 B (8–33 min), 30–100% (30–50 min); flow rate: 1 mL·min^−1^.

The encapsulation efficiency for the formulation prepared with 1:10 of extract/PMMA was determined using the previously validated UHPLC-UV-DAD method (Figure 2). Figure 2A shows the chromatogram of *P. edulis* extract, where the peak associated with isoorientin (**1**) appeared at a retention time of 27.0 min. Figure 2B shows the chromatogram of the aliquot withdrawn from the solution without NPEP obtained from the colloidal suspension, which did not show the isoorientin peak. Therefore, it can be inferred that 100% of isoorientin was encapsulated in the NPEP system, which corresponded to a final concentration of 538 µg/g (encapsulated isoorientin expressed as microgram per gram of NPEP).

A previous study reported high encapsulation rates for Eudragit-based nanoparticles (93%) and nanospheres (98.7%) containing *Curcuma longa* [18]. High encapsulation efficiency (80.88%) of the flavonoid dihydromyricetin into Eudragit nanoparticles was also reported in another study [24]. In most cases, the higher the lipophilicity of the molecule to be encapsulated, the higher the encapsulation efficiency [51,52]. On the other hand, the nano-encapsulation procedure described in this current study required the use of an organic phase composed of a mixture of ethanol and acetone. This method assures the dissolution of the natural compounds present in the hydroethanolic extract. In addition, smaller nanospheres with high encapsulation efficiency could be obtained using the traditional emulsification with solvent evaporation technique [22,23,24,25]. The ethanol/acetone ratio in the organic phase as well as the drug/polymer proportion play a critical role in the drug loading, particle size, and particle size distribution, as well as in the physical stability of the nano-formulation [53].

Plant extracts or their enriched fractions are complex mixtures whose solubility directly influence the nano-encapsulation procedure. In the present study, the hydroethanolic extract of *P. edulis* was successfully encapsulated into Eudragit nanoparticles using the nanoprecipitation method. In the case of hydrophobic extracts, such as that of *Piper cabralanum*, a further microemulsion procedure was necessary in order to obtain PMMA-loaded particles smaller than 60 nm [23].

On the basis of the high encapsulation efficiency of dihydromyricetin flavone [24] and quercetin flavonol [54] in Eudragit nanoparticles, in addition to the results of this current study, it seems likely to infer that the presence of flavonoids in *P. edulis* extract may interact with Eudragit copolymer and therefore preserve the cationic characteristics of the blank nanoparticles, corroborating the importance of electrostatic interaction between copolymer and flavonoids for a high encapsulation efficiency.

#### 3.2.2. Attenuated Total Reflectance Fourier Transform Infrared (ATR-FTIR)

**Figure 3:**
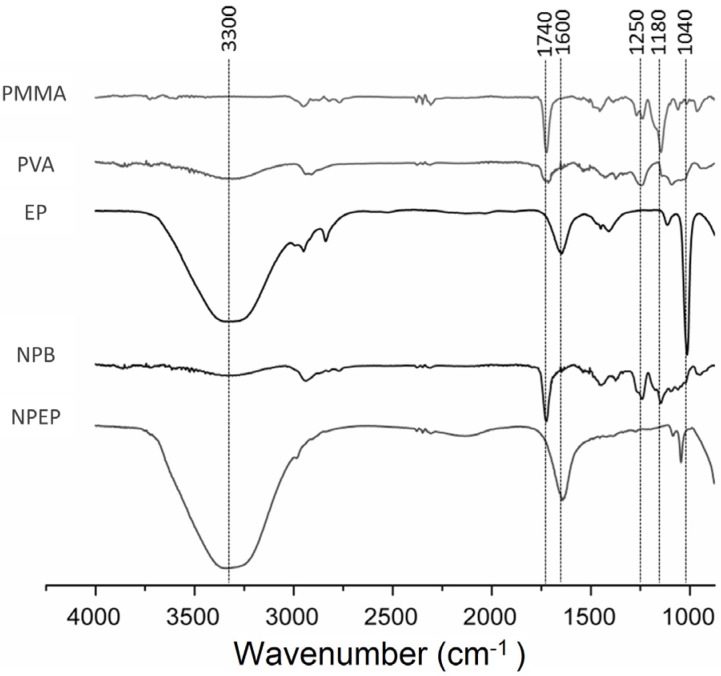
Attenuated total reflectance Fourier transform infrared (ATR-FTIR) spectra for each individual compound: hydroethanolic extract (EP), polyvinyl alcohol (PVA), and Eudragit polymethylmethacrylate (PMMA), blank nanoparticles (NPB), and loaded nanoparticles (NPEP). Specific bands of functional groups involved in the interaction of PVA, Eudragit PO, and EP are highlighted.

Figure 3 presents the ATR-FTIR spectra of Eudragit PMMA, PVA, and EP extracts, as well as NPB and NPEP at the extract/Eudragit ratio of 1:10. The ATR-FTIR spectrum of PVA showed a band at 3200 cm^−1^ due to hydroxyl groups and another at around 1050 cm^−1^, which is attributed to the primary alcohol groups. The Eudragit PMMA spectrum showed a band at 2900 cm^−1^ that corresponded to C–H bonds of aliphatic carbon, as well as the bands at 1750 cm^−1^, 1600 cm^−1^, and 1450 cm^−1^, which are assigned to the ester groups, to aromatic carbons, and CH_2_ groups, respectively [25,26]. These bands were also observed in the NPB spectrum, which confirms these components as constituents of the nanoparticles.

The spectrum of the extract of *P. edulis* shows an intense and broad band at 3300 cm^−1^ indicating the presence of OH groups, which is characteristic of flavonoids, phenolic acids, and other phenolic derivatives. The bands observed between 2800 and 3000 cm^−1^ are characteristic of stretching C–H bonds of sp^3^ carbon, indicating the presence of long unsaturated chains. Bands around 1600 cm^−1^ characterized aromatic compounds (C=C) and saponins (C=O). The band at 1180 cm^−1^ indicated the presence of nitrogen compounds such as alkaloids and cyanogenic glycosides (C–N), whereas the band at 1040 suggested stretches of C–O bonds of fatty acids, triterpene (C–O) esters, and polysaccharides [55].

The elongation of some of the bands in the spectrum of NPEP, especially those attributed to flavonoids at 3300 and 1600 cm^−1^, seemed to indicate some interaction between Eudragit and the phytocompounds present in the extract. In addition, a clear shift of the ester band of Eudragit (1740 cm^−1^) was observed in the spectrum of NPEP, which overlapped with the band at 1600 cm^−1^, assigned to aromatic C=C from flavonoids. The decrease in the band at 1040 cm^−1^ suggested a further interaction of long chain fatty acids and nitrogen compounds of cyanogenic glycosides with Eudragit. Thus, the experimental ATR-FTIR data confirmed that some chemical interaction took place between the phytocompounds from the *P. edulis* extract and Eudragit, which had been previously hypothesized on the basis of the relationship between the cationic nature of the copolymer and the high encapsulation efficiency [55].

#### 3.2.3. Atomic Force Microscopy (AFM)

**Figure 4:**
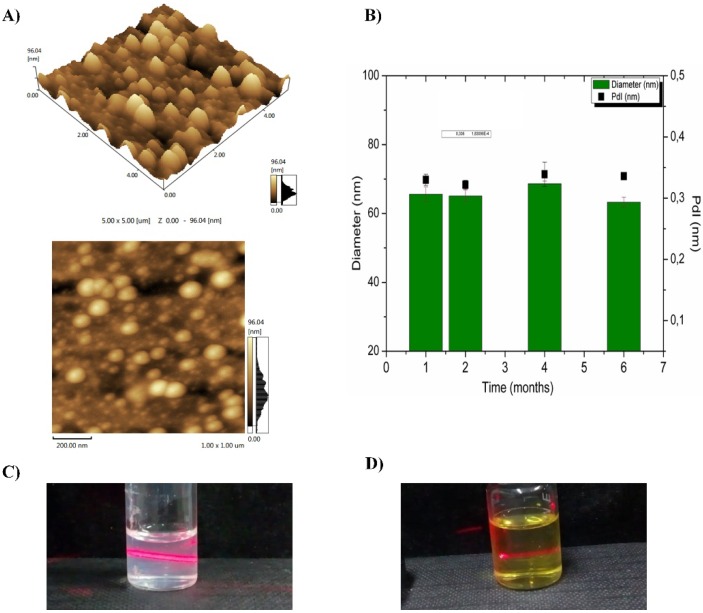
Atomic force microscopy (AFM) images of NPEP nanoparticles in 2D and 3D (**A**); mean diameter and zeta potential as a function of storage time for NPB and NPEP nanoparticles (1:10) during 6 months of storage (**B**); the red light scattering in the respective images highlights the “Tyndall effect” in NPB (**C**) and NPEP nanoparticles (**D**).

The AFM images assessed morphological aspects such as the shape and the surface of the particles. The AFM images of the nanoparticles prepared with the extract/Eudragit ratio of 1:10 were obtained in 2D and 3D (Figure 4A). This particular ratio was chosen on the basis of the previous results of particle size, zeta potential, and encapsulation efficiency. Both extract and nanoparticles showed slightly spherical shapes with smooth and uniform surfaces.

#### 3.2.4. Physicochemical Stability

The physicochemical stability of the formulations was investigated with the purpose of assessing whether anytime-dependent change occurred in the nanoparticles. The stability of both blank and NPEP was evaluated over a period of 6 months (Figure 4B). At this time interval, variations in the particle size and zeta potential were monitored. No considerable change in these parameters was observed for both formulations. It is worth pointing out that colloidal nanoparticles smaller than 100 nm exhibit high Brownian motion, which is detected in dynamic light scattering (DLS) measurements and correlated with the high translational diffusional coefficient. These systems are thermodynamically unstable and additional studies are needed in order to find the best composition and experimental parameters to obtain the most stable nanoparticles. In this study, the nanoparticles prepared with the EP/Eudragit ratio of 1:10 showed no significant changes in the evaluated parameters within the course of 6 months (Figure 4B). Moreover, blank nanoparticles (Figure 4B) and extract-loaded nanoparticles (Figure 4D) produced translucid dispersions that presented a Tyndall effect. The reasonable stability of both NPB and NPEP seemed to be due to both the high zeta potential and the steric stabilization of PVA as a surfactant. These factors may have induced the repulsion of the particles and therefore created a solvation layer around them, which prevented flocculation [24,26,54].

Regarding the non-encapsulated extract, it was possible to verify that under the same conditions of storage the sample lost its stability after 14 days, which was represented by the microbial contamination verified through the high turbidity and changes of color and smell [19]. Thus, the comparison between the stability of EP and NPEP indicates that the nano-encapsulation process improved the microbial stability of *P. edulis* extract. This is very advantageous, especially when we take into consideration the fact that previous reports have already demonstrated the poor microbial stability of this extract [6].

### 3.3. In Vivo Biocompatibility Study

The biocompatibility study was conducted in two stages. The first stage (48 h) assessed the behavioral changes and the animal’s general appearance. The purpose of the first stage was to establish the dose that would be used in the following stage. In stage 2 (14 days), the main study was conducted, which comprised observation, biochemical assays, and histopathologic analysis of the animals’ organs [38].

The observational study revealed that neither death nor sign of toxicity was observed for any of the doses evaluated for EP (5, 50, 300, and 2000 mg/kg), NPEP (10 mg/kg), and NPB (10 mg/kg-placebo) in the two stages of the study. The general appearance, gait, posture, and other behavior parameters remained unaltered during both stages. In addition, no significant variation in the biochemical parameters was observed (Table 4). After 14 days of administration of the samples, the biochemical assays AST, ALT, total protein, albumin, urea, and creatinine were all within the normal range for all animals.

**Figure 5:**
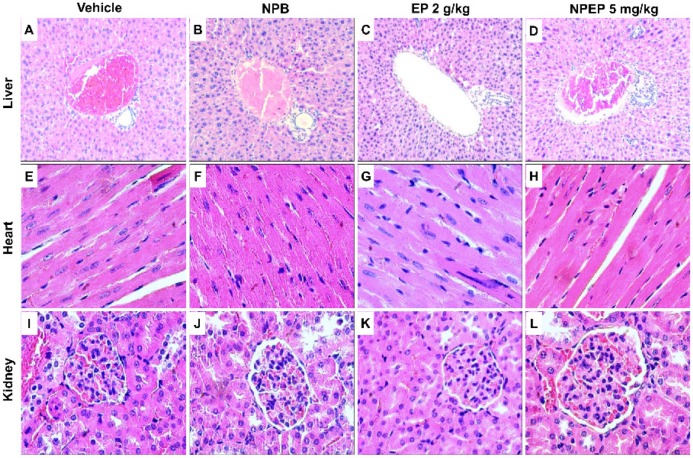
Histological analysis of organs of the in vivo biocompatibility study from the animals that received NPB, EP, and NPEP. Notes: the figures (**A**–**D**) show portal triad area of the liver without changes in both groups, (**E**–**H**) heart without changes in both groups, and (**I**–**L**) kidney renal glomerulus showing usual aspects. Scale bar: 400×.

Histopathological evaluation of organs such as brain, kidney, liver, spleen, and heart showed normal architecture and no abnormality in both control and treated groups (EP, NPEP, and NPB), as shown in Figure 5. The relative weight of the organs of the treated group were not different from those of the control group (Table 5). These results seem to indicate the absence of toxicity of *P. edulis* leaf extract [56,57], as well as the biocompatibility of NPB and NPEP [25,26].

### 3.4. In Vivo Behavioral Tests

**Figure 6:**
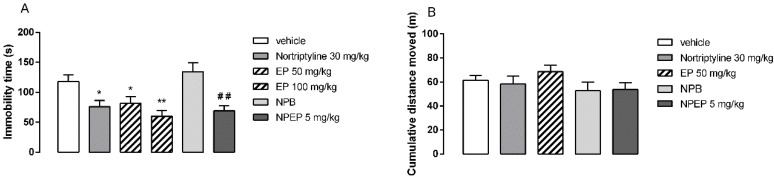
The effects of the administration of leaf extract (EP) (50; 100 mg/kg, administered orally (p.o.)) and extract-loaded nanoparticles (NPEP) (5 mg/kg, p.o.) of *Passiflora edulis* f. *flavicarpa* leaf, as well as blank nanoparticles (NPB) and positive control nortriptyline (30 mg/kg, p.o.) compared with negative control (vehicle) on the immobility time of mice during the forced swimming test (**A**) and on the accumulated distance moved during 30 min in the open field test (**B**). Results are represented as mean ± standard error of the mean (SEM) of 8–10 animals per group. * *p* < 0.05, ** *p* < 0.01 versus vehicle, one-way ANOVA, and Newman–Keuls’ test; ## *p* < 0.01 versus NPB and Student’s *t*-test.

The animals from the control group spent an average of 118 s of immobility during the forced swimming test. As shown in Figure 6A, the administration of the positive control nortriptyline (30 mg/kg, p.o.; *p* < 0.05), as well as EP extract (50 and 100 mg/kg, p.o.; *p* < 0.05 and < 0.01) and NPEP nanoparticles (5 mg/kg, p.o.; *p* < 0.01) significantly reduced the immobility time compared to that of the untreated mice. In fact, EP extract (50 mg/kg, p.o.) and NPEP nanoparticles (5 mg/kg, p.o.) exhibited a significant reduction in the immobility time at around 30.4% and 44.8%, respectively. Figure 6B illustrates the effects of EP extract, NPEP, and NPB in the spontaneous locomotor activity. The administration of EP extract, NPEP, and NPB did not modify the locomotor activity, thus indicating that the antidepressant effect of EP extract and NPEP nanoparticles, assessed in the forced swimming test, was not biased by the locomotor activity. Preliminary studies showed that doses below 50 mg/kg from EP extract did not show antidepressant activity.

Taken altogether, these findings suggest that the antidepressant-like effect of the extract-loaded obtained from the *P. edulis* f. *flavicarpa* was similar to that of nortriptyline (30 mg/kg, p.o.). In addition, such an effect was not influenced by the excipients used in the formulation, as NPB did not cause any change in the animals’ behavior, as assessed in the forced swimming and open field tests. Moreover, the antidepressant effect of NPEP (5 mg/kg) was 10-fold more potent than that of the free extract (i.e., EP 50 mg/kg).

A study previously carried out by our research group reported the acute antidepressant effect of the aqueous extract of *P. edulis* f. *flavicarpa* leaves after oral administration in mice (1000 mg/kg; p.o) [11]. The phytochemical analysis of the aqueous extract of *P. edulis* f. *flavicarpa* reported by Ayres et al. (2015) indicated the presence of secondary metabolites of flavonoids, which seems to be responsible for the antidepressant activity attributed to this extract [11]. Another study that investigated the acute administration of fractions of *P.edulis* f. *edulis* extract rich in these flavonoids also demonstrated the antidepressant effect in mice, suggesting the importance of these compounds in the pharmacological activity [2].

It is worth mentioning that antidepressant-like effects have been reported for isolated flavonoids, such as vitexin and orientin. In fact, acute administration of vitexin and chronic treatment with orientin induced antidepressant effects in rodent models of depression [3,17]. In silico studies showed the interaction of the flavonoids vicenin-2, vitexin, isovitexin, orientin, and isoorientin with monoamine oxidase enzyme. In fact, the inhibition of monoamine oxidase by these flavonoids was confirmed by in vitro enzymatic assays [15]. Such enzymes are responsible for monoamine metabolism, whose inhibition leads to an increase in catecholamines and monoamines in the synaptic cleft, therefore decreasing the depressive symptoms in humans [2,15,17].

As shown by Muniswamy et al., nanoparticles containing albumin-conjugated doxorubicin (134.4 ± 10.85 nm) were able to improve blood-brain barrier (BBB) penetration and enhance anticancer activity of doxorubicin, as assessed by in vitro cellular and ex vivo studies using a cultured cell monolayer of mouse cerebral endothelial cell line (bEnd3) [36]. In addition, de Papa et al. demonstrated the interaction between cationic PMMA nanoparticles (97 ± 0.065) and microglial cells. They concluded that the permeation of positively charged nanoparticles across microglial cells was significantly higher than that of negatively charged nanoparticles [21].

Previous reports show that flavonoid encapsulation using Eudragit copolymer promotes the highest degree of encapsulation of these natural compounds [24,54]. In our current study, NPEP showed similar characteristics including the chemical interactions between the flavonoid derivatives from *P. edulis* extract and Eudragit nanoparticles [24,54]. The physicochemical characterization of NPEP indicated the successful encapsulation of the flavonoids extracted from *P. edulis* leaves into Eudragit nanoparticles. Moreover, this encapsulation markedly improved the antidepressant activity of the extract (a 10-fold increase in comparison with the free extract). Furthermore, according to previous findings [3,15,16,17], it seems that the antidepressant activity of NPEP is attributed to vicenin-2, vitexin, isovitexin, orientin, and isoorientin, suggesting a monoaminergic mechanism for their activity in the central nervous system [2,5,11,15].

## 4. Conclusions

In this study, *P. edulis* f. *flavicarpa* leaf extract was used as a promising alternative to treat depression. The encapsulation process considerably enhanced the pharmacological activity of *P. edulis* f. *Flavicarpa* extract. The small-sized cationic nanoparticles were able to encapsulate the extract with high efficiency and stability. These properties might have contributed to better targeting the flavonoids towards the central nervous system of the treated mice. The experimental data discussed in this study provided theoretical and practical knowledge to better understand the pharmacological potential of the leaf extract of *P. edulis* f. *flavicarpa*. Further in vivo acute and chronic studies with extract-loaded nanoparticles or its association with synthetic antidepressant drugs could reveal promising therapeutic alternatives to treat depression.

## Figures and Tables

**Table 1:** Linearity, limit of detection (LOD), and limit of quantification (LOQ) of the LC-QqQ-MS/MS analytical method for the quantification of flavonoids in the samples.

Compounds	Linearity Range (ng·mL^−1^)	Calibration Equation	Correlation Factor (*r*)	LOD (ng·mL^−1^)	LOQ (ng·mL^−1^)
Vicenin-2	5–500	*y* = 136.2*x* − 136.2	0.9960	1.66	5.0
Orientin	5–500	*y* = 115.47*x* − 539.0	0.9973	1.66	5.0
Isoorientin	5–500	*y* = 98.41*x* − 162.4	0.9993	1.66	5.0
Vitexin	5–500	*y* = 340.0*x* − 501.0	0.9984	1.00	3.0
Isovitexin	5–500	*y* = 264.94*x* + 667.0	0.9986	1.00	3.0

**Table 2:** Precision and accuracy levels of the LC-QqQ-MS/MS analytical method for the quantification of flavonoids in the samples.

Compounds	Concentration	Precision	Accuracy (Recovery)	
	(ng·mL^−1^)	RSD (%)	(ng·mL^−1^)	SRE (%)
Vicenin-2	10	3.7	10.1	1.0
	50	1.8	51.9	3.9
	100	3.4	101.0	1.0
Orientin	10	3.7	10.0	3.1
	50	1.8	52.2	−1.4
	100	3.4	102.1	0.5
Isoorientin	10	3.7	10.3	−1.9
	50	1.8	49.3	−0.8
	100	3.4	100.5	0.2
Vitexin	10	1.9	10.2	1.6
	50	2.6	51.1	2.2
	100	3.4	103.4	3.4
Isovitexin	10	1.5	9.8	−1.9
	50	3.5	49.6	−0.8
	100	2.4	100.2	0.2

**Table 3:** Physicochemical properties of the extract-loaded nanoparticles.

Extract/Eudragit Ratio	Diameter (nm) ± SD	PdI (nm) ± SD	Zeta Potential (mV) ± SD
0	106.2 ± 1.2	0.245 ± 0.03	+35.7 ± 1.3
1:10	65.6 ± 2.1	0.330 ± 0.01	+38.4 ± 1.2
1:5	90.7 ± 0.5	0.305 ± 0.01	+40.4 ± 2.2
1:2.5	128.8 ± 0.2	0.129 ± 0.01	+37.9 ± k0.4

PdI (polydispersity index), nm (nanometer), standard deviation (SD), millivolt (mV).

**Table 4:** Biochemical parameters analyzed in the biocompatibility study of animals treated with *P. edulis* leaf extract as well as loaded into Eudragit nanoparticles.

Analyte	Vehicle	NPB ^1^	EP ^1^ (2 g/kg)	NPEP ^1^ (5 mg/kg)
**Liver Function**				
Total protein	5.37 ± 0.29	5.00 ± 0.53	5.29 ± 0.43	4.79 ± 0.28
Albumin	2.28 ± 0.11	1.97 ± 0.09	2.30 ± 0.15	1.96 ± 0.05
ALT ^2^	48.14 ± 10.07	37.40 ± 1.14	47.20 ± 8.50	37.4 ± 7.47
AST ^2^	77.00 ± 11.65	95.20 ± 11.61	83.20 ± 11.20	70.20 ± 13.14
**Kidney Function**				
Urea	42.86 ± 10.79	38.8 ± 8.5	52.60 ± 6.5	43.4 ± 10.92
Creatinine	0.25 ± 0.07	0.40 ± 0.12	0.23 ± 0.06	0.33 ± 0.05

^1^ NPB: blank nanoparticles, EP: leaf extract, NPEP: extract-loaded nanoparticles (*n* = 5). ^2^ ALT: aminotransferase; AST: aspartate aminotransferase.

**Table 5:** Relative organ weight and weight variation in mice that received oral administration of blank nanoparticles, extract, and extract-loaded nanoparticles of *P. edulis*.

Organs	Vehicle	NPB ^1^	EP ^1^ (2 g/kg)	NPEP ^1^ (5 mg/kg)
Brain	0.0126 ± 0.0003	0.0135 ± 0.0020	0.0128 ± 0.0005	0.0136 ± 0.0007
Liver	0.0518 ± 0.0030	0.0424 ± 0.0028	0.0484 ± 0.0116	0.0424 ± 0.0028
Heart	0.0055 ± 0.0008	0.0059 ± 0.0009	0.0064 ± 0.0014	0.0064 ± 0.0014
Spleen	0.0038 ± 0.0006	0.0044 ± 0.0016	0.0042 ± 0.0006	0.0041 ± 0.0010
Kidneys	0.0135 ± 0.0018	0.0104 ± 0.0008	0.0107 ± 0.0035	0.0111 ± 0.0011
Weight variation	1.02 ± 0.0206	1.04 ± 0.0715	1.01 ± 0.0154	1.01 ± 0.0247

^1^ NPB: blank nanoparticles, EP: leaf extract, NPEP: extract-loaded nanoparticles (*n* = 5).

## Supplementary Materials

The following are available online at https://www.mdpi.com/1999-4923/12/4/383/s1: Figure S1: Calibration curve data obtained for vicenin-2 using HPLC-QqQ-MS/MS. Figure S2: Calibration curve data obtained for orientin using HPLC-QqQ-MS/MS. Figure S3: Calibration curve data obtained for isoorientin using HPLC-QqQ-MS/MS. Figure S4: Calibration curve data obtained for vitexin using HPLC-QqQ-MS/MS. Figure S5: Calibration curve data obtained for isovitexin using HPLC-QqQ-MS/MS. Figure S6: Calibration curve data obtained for isoorientin by UHPLC-UV-DAD. Table S1: Validation data obtained for accuracy, precision, as well as limits of detection and quantification for isoorientin using UHPLC-UV-DAD. Figure S7: UHPLC-UV-DAD chromatogram of supernatant from NPEP for encapsulation efficiency evaluation (replicate 1). Figure S8: UHPLC-UV-DAD chromatogram of supernatant from NPEP for encapsulation efficiency evaluation (replicate 2).
