# Supplementary material for: In Vivo Antidepressant Effect of Passiflora edulis f. flavicarpa into Cationic Nanoparticles: Improving Bioactivity and Safety
Source: Pharmaceutics. 2020 Apr 21;12(4):383. doi: 10.3390/pharmaceutics12040383 (PMC7238140; doi:10.3390/pharmaceutics12040383)
Supplement: Supplementary file 1 [file pharmaceutics-12-00383-s001.pdf]

pharmaceutics

Supplementary Materials: In Vivo Antidepressant Effect of *Passiflora edulis* f. *flavicarpa* into Cationic Nanoparticles: Improving Bioactivity and Safety

Jovelina Samara Ferreira Alves, Alaine Maria dos Santos Silva, Rodrigo Moreira da Silva, Pamella Rebeca Fernandes Tiago, Thais Gomes de Carvalho, Raimundo Fernandes de Araújo Júnior, Eduardo Pereira de Azevedo, Norberto Peporine Lopes, Leandro De Santis Ferreira, Elaine Cristina Gavioli, Arnóbio Antônio da Silva-Júnior and Silvana Maria Zucolotto

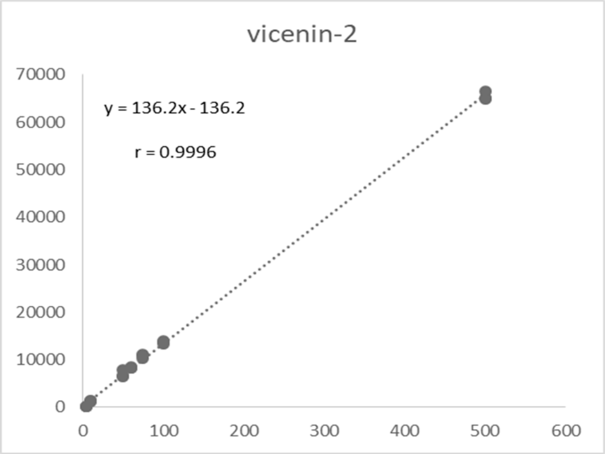

Figure S1. Calibration curve data obtained for the vicenin-2 using HPLC-QqQ-MS/MS.

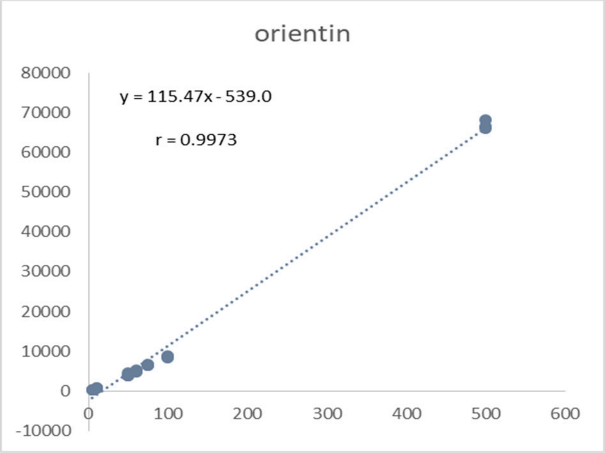

Figure S2. Calibration curve data obtained for the orientin using HPLC-QqQ-MS/MS.

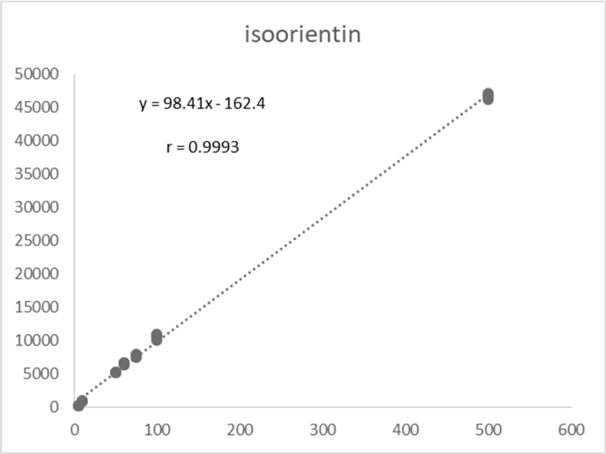

Figure S3. Calibration curve data obtained for the isoorientin using HPLC-QqQ-MS/MS.

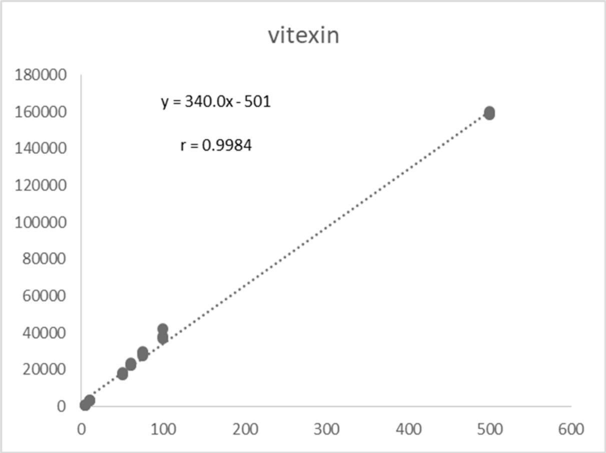

Figure S4. Calibration curve data obtained for the vitexin using HPLC-QqQ-MS/MS.

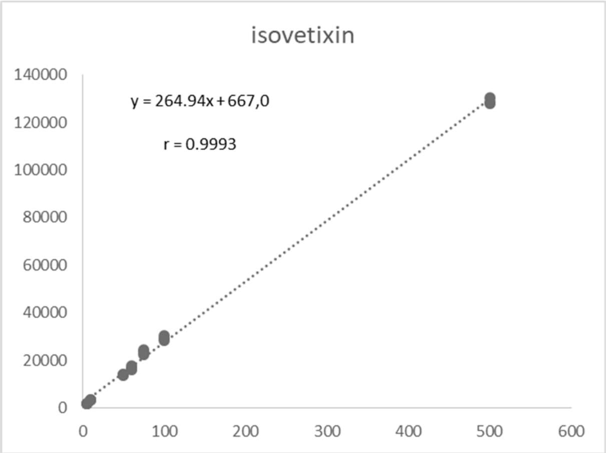

Figure S5. Calibration curve data obtained for the isovitexin using HPLC-QqQ-MS/MS.

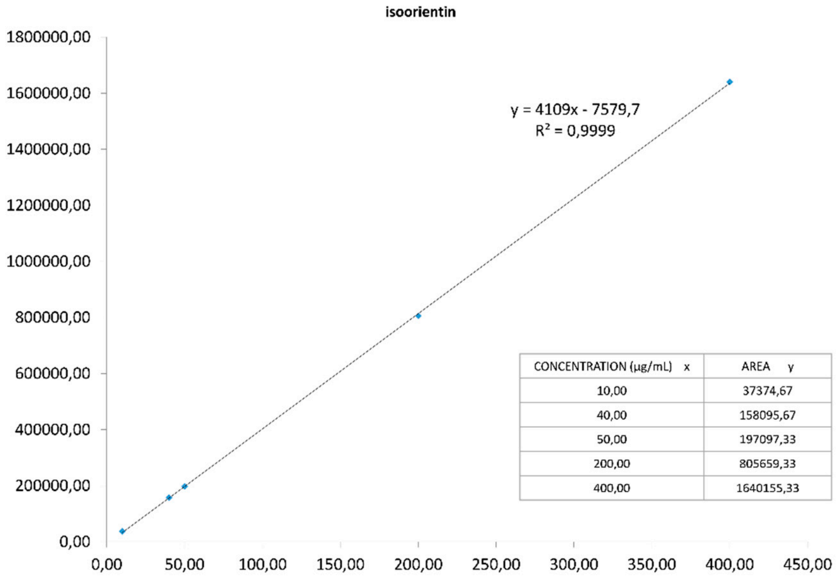

Figure S6. Calibration curve data obtained for isoorientin by UHPLC-UV-DAD for encapsulation efficiency evaluation.

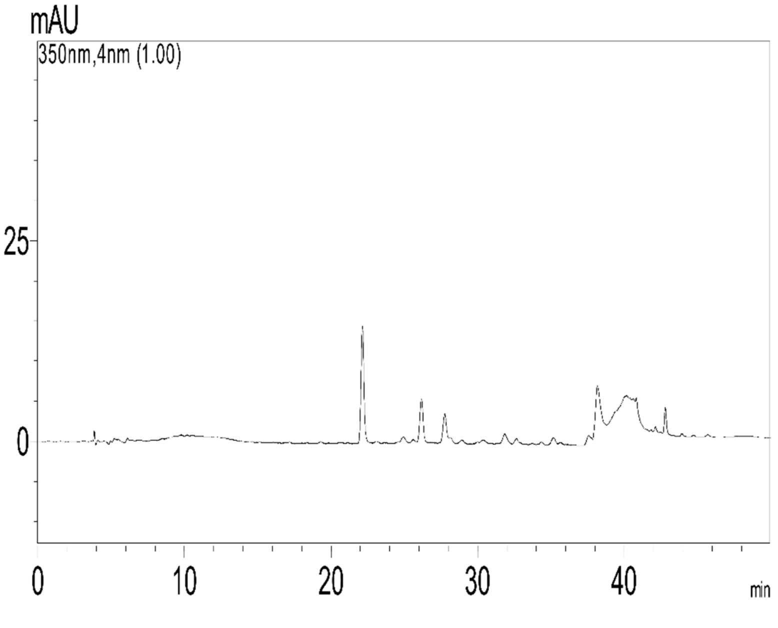

Figure S7. UHPLC-UV-DAD Chromatogram of supernatant from NPEP from encapsulation efficiency evaluation (replicate 1).

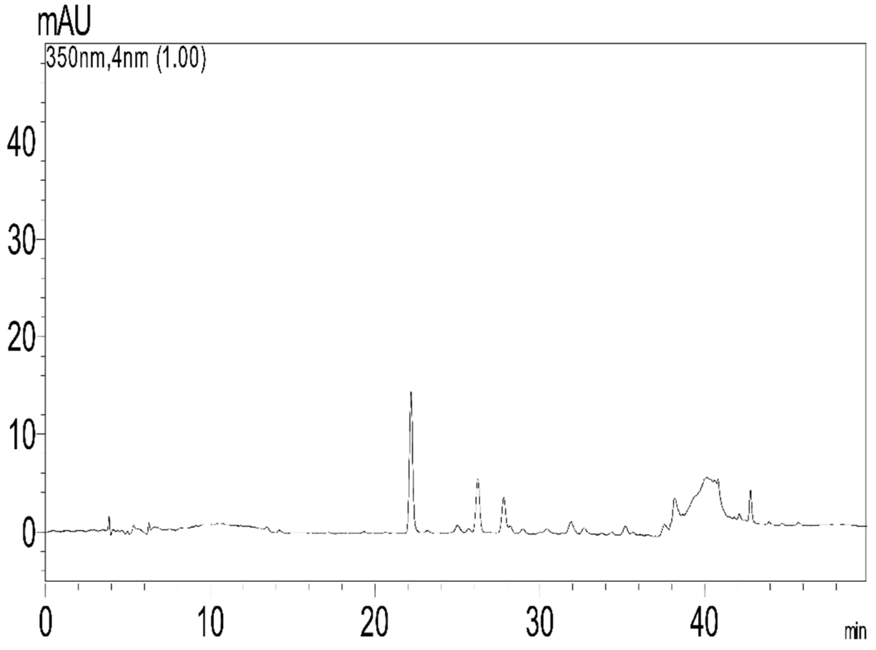

Figure S8. UHPLC-UV-DAD Chromatogram of supernatant from NPEP from encapsulation efficiency evaluation (replicate 2).

Table S1. Validation data obtained for the accuracy, precision and detection and quantification limits of isoorientin compound using UHPLC-UV-DAD.

**Linearity range (μg/mL)**	**Calibration equation**		**Correlation factor (r^2)**	**LOD (μg/mL)**	**LOQ (μg/mL)**
**10-400**	y=4109x-7579.7		0.9999	1.0	5.0
**Repeatability**		**Intermediate precision**		**Accuracy (recovery[a])**	
**Concentration (μg/mL)**	**RSD (%)**	**Concentration (μg/mL)**	**RSD (%)**	**Mean (%)**	**RSD (%)**
50	1.660	50	0.978	91.00	1.780
200	0.153	200	0.209	97.69	0.996
400	0.271	400	1.010	98.18	0.135
